# Functional and Biochemical Endothelial Profiling *In Vivo* in a Murine Model of Endothelial Dysfunction; Comparison of Effects of 1-Methylnicotinamide and Angiotensin-converting Enzyme Inhibitor

**DOI:** 10.3389/fphar.2017.00183

**Published:** 2017-04-10

**Authors:** Anna Bar, Mariola Olkowicz, Urszula Tyrankiewicz, Edyta Kus, Krzysztof Jasinski, Ryszard T. Smolenski, Tomasz Skorka, Stefan Chlopicki

**Affiliations:** ^1^Jagiellonian Centre for Experimental Therapeutics, Jagiellonian UniversityKrakow, Poland; ^2^Chair of Pharmacology, Jagiellonian University Medical CollegeKrakow, Poland; ^3^Department of Biochemistry, Medical University of GdanskGdansk, Poland; ^4^Department of Biotechnology and Food Microbiology, Poznan University of Life SciencesPoznan, Poland; ^5^Department of Magnetic Resonance Imaging, Institute of Nuclear Physics Polish Academy of SciencesKrakow, Poland

**Keywords:** endothelial function, atherosclerosis, MRI, 1-methylnicotinamide, perindopril, plasma angiotensin profile, L-Arg/ADMA ratio

## Abstract

Although it is known that 1-methylnicotinamide (MNA) displays vasoprotective activity in mice, as yet the effect of MNA on endothelial function has not been demonstrated *in vivo*. Here, using magnetic resonance imaging (MRI) we profile the effects of MNA on endothelial phenotype in mice with atherosclerosis (ApoE/LDLR^-/-^) *in vivo*, in comparison to angiotensin (Ang) -converting enzyme (ACE) inhibitor (perindopril), with known vasoprotective activity. On a biochemical level, we analyzed whether MNA- or perindopril-induced improvement in endothelial function results in changes in ACE/Ang II-ACE2/Ang-(1–7) balance, and L-arginine/asymmetric dimethylarginine (ADMA) ratio. Endothelial function and permeability were evaluated in the brachiocephalic artery (BCA) in 4-month-old ApoE/LDLR^-/-^ mice that were non-treated or treated for 1 month or 2 months with either MNA (100 mg/kg/day) or perindopril (10 mg/kg/day). The 3D IntraGate^®^FLASH sequence was used for evaluation of BCA volume changes following acetylcholine (Ach) administration, and for relaxation time (T_1_) mapping around BCA to assess endothelial permeability using an intravascular contrast agent. Activity of ACE/Ang II and ACE2/Ang-(1–7) pathways as well as metabolites of L-arginine/ADMA pathway were measured using liquid chromatography/mass spectrometry-based methods. In non-treated 6-month-old ApoE/LDLR^-/-^ mice, Ach induced a vasoconstriction in BCA that amounted to –7.2%. 2-month treatment with either MNA or perindopril resulted in the reversal of impaired Ach-induced response to vasodilatation (4.5 and 5.5%, respectively) and a decrease in endothelial permeability (by about 60% for MNA-, as well as perindopril-treated mice). Improvement of endothelial function by MNA and perindopril was in both cases associated with the activation of ACE2/Ang-(1–7) and the inhibition of ACE/Ang II axes as evidenced by an approximately twofold increase in Ang-(1–9) and Ang-(1–7) and a proportional decrease in Ang II and its active metabolites. Finally, MNA and perindopril treatment resulted in an increase in L-arginine/ADMA ratio by 107% (MNA) and 140% (perindopril), as compared to non-treated mice. Functional and biochemical endothelial profiling in ApoE/LDLR^-/-^ mice *in vivo* revealed that 2-month treatment with MNA (100 mg/kg/day) displayed a similar profile of vasoprotective effect as 2-month treatment with perindopril (10 mg/kg/day): i.e., the improvement in endothelial function that was associated with the beneficial changes in ACE/Ang II-ACE2/Ang (1–7) balance and in L-arginine/ADMA ratio in plasma.

## Introduction

1-Methylnicotinamide (MNA), the major metabolite of NA synthesized in the liver in the reaction involving NNMT, when given exogenously has distinct therapeutic activity, despite the fact, that it has long been considered inactive (Aksoy et al., 1994). Indeed, topical MNA alleviates the inflammatory responses in skin diseases such as acne, contact dermatitis or rosacea (Gebicki et al., 2003; Wozniacka et al., 2005). In turn, systemic administration of MNA exerts anti-thrombotic (Chlopicki et al., 2007), anti-inflammatory (Bryniarski et al., 2008), and gastroprotective (Brzozowski et al., 2008) properties mediated by the activation of COX-2 and PGI_2_ pathways. Furthermore, MNA can improve endurance exercise capacity in mice with diabetes, and may decrease the cardiovascular risk of exercise (Przyborowski et al., 2015). MNA was demonstrated to have hepatoprotective activity against concanavalin A-induced liver injury through the downregulation of IL-4 and TNF-α signaling (Sternak et al., 2010; Jakubowski et al., 2016), and to inhibit metastasis formation in a murine model of metastatic mammary gland cancer (4T1) in BALB/c mice (Blazejczyk et al., 2016). Interestingly, 1-MNA has also been shown to restore endothelial function in diabetic hypertriglycemic rats (Bartuś et al., 2008) analyzed *ex vivo* in isolated aorta, suggesting that the improvement in endothelial function may represent an important target of MNA activity and may explain therapeutic efficacy of MNA in various diseases including diabetes (Watała et al., 2009) and atherosclerosis (Mateuszuk et al., 2016). Indeed, in our recent study anti-atherosclerotic effects of MNA in ApoE/LDLR^-/-^ mice, including the inhibition of inflammatory burden in plaques, diminished systemic inflammation, diminished platelet activation that were associated with an improvement in PGI_2_ and NO-dependent endothelial function. These results support the notion that pronounced effects of MNA on endothelial function could contribute to therapeutic efficacy of MNA (Mateuszuk et al., 2016). However, in none of the previous experimental studies of MNA was endothelial function assessed *in vivo*, but only *in ex vivo* vascular preparation (Bartuś et al., 2008; Mateuszuk et al., 2016).

Endothelial dysfunction, being a consequence of vascular homeostatic imbalance is a hallmark of various cardiovascular diseases including atherosclerosis (Anderson et al., 1995), hypertension (Dharmashankar and Widlansky, 2010), heart failure (Marti et al., 2012), as well as non-cardiovascular diseases such as cancer (Franses et al., 2013). Vascular dysfunction is associated with activation of pro-inflammatory signaling molecules including adhesion molecules, chemokines and cytokines (Karbach et al., 2014; Steven et al., 2015). There is also evidence that ROS play an important role in vascular inflammation (Zhou et al., 2011) and tissue damage (Mittal et al., 2014). There are number of enzymatic sources of endothelial ROS, including eNOS uncoupling, converting beneficial NO synthase into a detrimental superoxide-producing enzyme (Karbach et al., 2014; Schulz et al., 2014).

Currently, many biochemical assays and functional tests are used for the evaluation of impaired NO-dependent function, oxidant stress and pro-inflammatory phenotype of endothelial dysfunction in humans. Prognostic value of biochemical measurements of endothelial biomarkers is still not widely accepted (Walczak et al., 2015). However, endothelial function measurements based on the assessment of impairment of NO-dependent vasodilatation in the coronary and peripheral circulation have prognostic importance in predicting adverse cardiovascular events (Schächinger et al., 2000; Halcox et al., 2002). In particular, a non-invasive endothelial function assessment, based on monitoring the brachial artery diameter with a two-dimensional ultrasound, before and after artery occlusion is considered a gold-standard method (Frolow et al., 2015). MRI has also successfully been used for non-invasive measurement of FMD in humans (Lee et al., 2007; Canton et al., 2012; Redheuil, 2014; Teixido-Tura et al., 2014), but studies of endothelial functional phenotype in mice *in vivo* are associated with greater technical challenges (Botnar and Makowski, 2012). Given high spatio-temporal resolution of MRI, it seems to be the method of choice to assess endothelium-dependent response in mice. Indeed, MRI-based tests could provide an excellent tool to gain better insight into endothelium-dependent mechanisms in experimental studies in mice (Gotschy et al., 2013; Herold et al., 2009). Phinikaridou et al. (2012) were the first to describe an MRI-based method for the noninvasive assessment of endothelial-dependent vasodilatation and permeability *in vivo* in mice, with the use of an albumin-binding magnetic resonance CA. In our recent studies, we developed a 3D MRI-based assessment of endothelium-dependent vasodilatation induced by acetylcholine (Ach) and endothelial permeability fully based on the use of retrospectively self-gated 3D gradient-echo sequence (Bar et al., 2016). The developed approach has been validated in two distinct murine models of endothelial dysfunction, including atherosclerotic (ApoE/LDLR^-/-^ mice) and HFD-fed mice, showing that our approach allows for a reliable detection of the impairment of NO-dependent vasodilatation and changes in endothelial permeability.

Genetically modified mice without apolipoprotein E and receptor for low density lipoprotein (mouse apolipoprotein E-deficient and Low-Density Lipoprotein Receptor-Deficient Double Knockout, ApoE/LDLR^-/-^), constitute reliable mouse model of endothelial dysfunction linked to atherosclerosis. In this strain of mice, spontaneously atherosclerosis develops without administration of atherogenic diet (Kostogrys et al., 2012), and endothelial dysfunction precedes the atherosclerotic plaque development (Csányi et al., 2012), similarly as it occurs in humans. In turn, the early development of endothelial dysfunction in single ApoE^-/-^ mice was not univocally accepted (Crauwels et al., 2003; Villeneuve et al., 2003).

Given that the assessment of the endothelial phenotype *in vivo* in a reliable mouse model of endothelial dysfunction is essential for the preclinical profiling of vasoprotective compounds, the aim of the present study was to take advantage of the 3D MRI-based method to profile effects of MNA on functional phenotype of endothelium in ApoE/LDLR^-/-^ mice *in vivo*, in comparison to standard vasoprotective treatment such as ACE-I (perindopril), that is known to display significant vasoprotective effect in various experimental models including atherosclerosis (Chłopicki and Gryglewski, 2005). We also analyzed whether MNA- or perindopril-induced improvement in endothelial function is associated with biochemical changes in plasma in ACE/Ang-II-ACE2/Ang-(1–7) balance and L-Arg/ADMA ratio.

## Materials and Methods

### Animals

In this study, 4-month-old ApoE/LDLR^-/-^ mice (body weight of 20–30 g), bred in the Department of Human Nutrition, University of Agriculture (Krakow, Poland), were transported to the animal house at Jagiellonian Centre for Experimental Therapeutics (JCET), Jagiellonian University (Krakow, Poland) and were treated for 1 or 2 months with MNA (syntethized by dr Jan Adamus, Technical University in Lodz, Poland: 100 mg/kg/day in diet) or perindopril (a gift from Servier, Warszawa Poland: 10 mg/kg/day in drinking water). Doses of compounds used in this study were based on our previous published (Chlopicki et al., 2007; Mateuszuk et al., 2016) and unpublished studies, for MNA and perindopril, respectively. Two groups of 4-month-old ApoE/LDLR^-/-^ mice were left untreated (as control groups) for 1 and 2 months, respectively. The sizes and names of the experimental groups are reported in the legends of the corresponding graphs. All mice were bred in standard conditions (LD: 12/12, humidity: 60%, temperature: 23°C), and housed in pathogen-free conditions. Prior to the MRI experiment, the animals were transported to the animal house at the Institute of Nuclear Physics (Krakow, Poland) to assess endothelial phenotype *in vivo.* After *in vivo* measurements, mice were sacrificed in order to collect blood. The experimental design is illustrated in Supplementary Figure S1. All experiments were approved by the Ethics Local Committee of Jagiellonian University (Krakow, Poland) and were compliant with the Guide for the Care and Use of Laboratory Animals of the National Academy of Sciences (NIH publication No. 85–23, revised 1996).

### *In Vivo* Assessment of NO-dependent Endothelial Response to Ach and Endothelial Permeability by MRI

MRI experiments were performed using a 9.4T scanner (BioSpec 94/20 USR, Bruker, BioSpin GmbH, Germany), as described previously (Bar et al., 2016). Mice were anaesthetized using isoflurane (Aerrane, Baxter Sp. z o. o., Warszawa, Poland, 1.7 vol. %) in an oxygen and air (1:2) mixture. Body temperature was maintained at 37°C using circulating warm water. ECG, respiration and body temperature were monitored using a Model 1025 Monitoring and Gating System (SA Inc., Stony Brook, NY, USA). Mice were imaged in the supine position to test the vasomotor response of the vessel. 3D images of the aortic arch prior to and 25 min after intraperitoneal Ach administration (Sigma–Aldrich, Poznan Poland: 50 μl, 16.6 mg/kg) were acquired using the cine IntraGate^TM^ FLASH 3D sequence and reconstructed with the IntraGate 1.2.b.2 macro (Bruker, BioSpin GmbH, Germany). T_1_ maps were measured before and 30 min after intravenous administration of albumin-binding gadolinium CA (Galbumin, BioPal, Worcester, MA, USA – 25 mg/ml, 4.5 ml/kg) using the VFA technique (Cheng and Wright, 2006; Wang et al., 2006), by sampling the signal, using varying values of flip angles and then fitting the result to an expected T_1_-dependent signal model. Imaging parameters for endothelial function assessment included the following: repetition time (TR) – 6.4 ms, echo time (TE) – 1.4 ms, field of view (FOV) – 30 mm × 30 mm × 5 mm, matrix size – 256 × 256 × 30, flip angle (FA) – 30°, and number of accumulations (NA) – 15, reconstructed to seven cardiac frames. Total scan time was 10 min. Imaging parameters for endothelial permeability assessment included the following: TR – 10 ms, TE – 1.1 ms, FOV – 30 mm × 30 mm × 4 mm, matrix size – 192 × 160 × 8, number of repetitions – 12, and reconstructed to one cardiac frame. Eight FA were used: 2°, 4°, 6°, 8°, 14°, 20°, 30°, 50°. FA values were set by changing the length of a radiofrequency pulse, with constant amplifier power. Total scan time for all angles was 16 min. The experimental design is illustrated in Supplementary Figure S1.

### MRI Data Analysis

**FIGURE 1:**
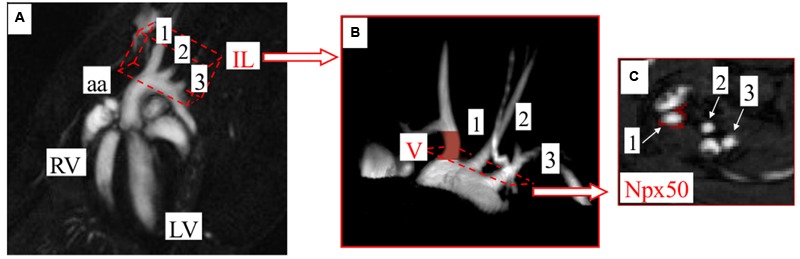
**Methodology to assess endothelium-dependent response and changes in endothelial permeability in brachiocephalic artery (BCA) *in vivo* by MRI. (A)** Coronal view of the heart. Image showing position of the imaging layer (IL) used for 3D imaging of the aortic arch. **(B)** 3D image of aortic arch acquired with the cine IntraGate^®^ FLASH 3D sequence. Endothelial function assessment, expressed as changes in the vessels volume (V) can be performed in vessels arising from aortic arch. The end-diastolic volumes of BCA were assessed prior to and 25 min after intraperitoneal Ach administration. **(C)** Representative images of vessels cross-sections, in which a number of pixels, for which T_1_ had changed more than 50% after CA administration (Npx50), is marked in red. BCA is indicated as 1, left carotid artery (LCA) as 2, left subclavian artery (LSA) as 3. RV, right ventricle; LV, left ventricle.

Time-resolved 3D images of the aortic arch (**Figure 1A**) were analyzed to endothelial function assessment in BCA, using ImageJ software 1.46r (NIH Bethesda, MD, USA) and scripts written in Matlab (MathWorks, Natick, MA, USA). Images were reconstructed to seven cardiac frames and imported into ImageJ as a hyperstack (**Figure 1B** matrix: 256 × 256, slices: 30, frames: 7). Further analysis was performed in diastole of BCA, using small hyperstack (**Figure 1B** marked in red color matrix: 256 × 256, slices: 5, frames: 1) starting at the base of the vessel and ending just before the branch. Cross-sectional areas of BCA at each slices were obtained using thresholding segmentation and exported to Matlab, where BCA volumes were reconstructed and calculated. Detailed analysis was described in Supplementary Material of our previous work (Bar et al., 2016). In order to endothelial permeability assessment, obtained images were used to calculate the T_1_ around the BCA lumen before and after CA administration. The signal model was fitted pixel by pixel using Matlab software developed in house. Two T_1_ maps (before and after CA administration) were compared, using scripts written in Matlab, and pixels for which T_1_ had changed significantly (by more than 50%) after CA administration were marked in red (**Figure 1C**). The threshold value (50%) was determined experimentally. All red pixels were counted by program as number of pixels, for which T_1_ had changed more than 50% after CA administration (Npx50).

### Measurement of Ang Profile in Plasma

The blood was drawn from the heart, collected in tubes containing heparin (20 units/ml) and immediately mixed with protease inhibitor cocktail (Sigma–Aldrich, Poznan, Poland) in a ratio of 19:1 (v/v), and centrifuged at 1,000 × *g* for 10 min to isolate plasma. Afterward, the resulting plasma was transferred into Protein LoBind tubes (Eppendorf, Hamburg, Germany), split into aliquots and stored frozen at -80°C until analysis.

Protein precipitation with ACN was used as the sample pre-treatment method to remove high molecular plasma compounds. CS, QC samples, and RS were created by spiking 25 μl (pooled blank – CS, QC) plasma with 20 μl appropriate working standard solution or water (RS) and 5 μl IS – [Asn^1^, Val^5^]-Ang II (2,500 pg/ml). Calibration curve standards were made at: 10; 50; 100; 250; 300; 400; 500 pg/ml (IS – 250 pg/ml) concentrations, respectively. CS at seven different concentration levels or adequate QC and RS were deproteinized with ACN (in 4:1 (v/v) proportion to the samples used) and assayed.

The LC-MS/MS analyses of plasma samples were performed on an UltiMate 3000 Rapid Separation nano-LC system (Dionex, Thermo Scientific, San Jose, CA, USA) interfaced via a ChipMate^TM^ nanoelectrospray ion source (Advion, USA) to a TSQ Vantage triple quadrupole mass spectrometer (Thermo Scientific). The samples were injected in 5 μl aliquots onto a trapping column for desalting and concentrating of the analytes which in the next step was switched in-line with the separation C18 column to elute the peptides at a flow rate of 300 nl/min with an increasing percentage of the organic solvent. Separation was accomplished using a gradient of phase A (acetic acid (1%, v/v) in H_2_O) and phase B (acetic acid (1%, v/v) in ACN) as follows: 2% B for 5 min, 2–98% B for 15 min, and 98% B for 5 min. The mass spectrometer was operated in the positive ion mode and the detection of Ang peptides in MRM mode was performed. The two most sensitive/specific ion transitions were measured for each of the 9 Ang peptides determined [Ang I, II, III, IV, (1–7), (1–9), Ang A, alamandine and Ang-(1–12)]. Analytical method details as well as the MS/MS transitions monitored in the protocol are described elsewhere (Olkowicz et al., 2016). Instrument control, data collection, and analysis were achieved with the Thermo Xcalibur (version 2.1) software.

### Measurement of Flux Through L-Arg Metabolic Pathways

Plasma amino acid concentrations were determined using a Surveyor HPLC system (Thermo Scientific) coupled to a TSQ Vantage Triple-Stage Quadrupole mass spectrometer, as described recently (Smolenska et al., 2016). Briefly, an aliquot of plasma (50 μl) was deproteinized with 100 μl of ACN maintained in ice. The tubes were then centrifuged at 4°C, 16,000 × *g* for 10 min and the resultant supernatant was collected and subjected to freeze-drying. The residue was next reconstituted with 50 μl of H_2_O acidified with 0.1% (v/v) FA and analyzed using an LC/MS system as detailed below.

Chromatographic separation was achieved using a Synergi Hydro-RP column (50 mm × 2.0 mm i.d., 2.5 μm) fitted with a security guard (Phenomenex, Torrance, CA, USA). The mobile phase consisted of a mixture of phase A (5 mM NFPA in water) and phase B (0.1% (v/v) FA in ACN), that was delivered at 0.2 ml/min in a gradient from 0 to 45% B in 5.5 min. The effluent of the HPLC column was directed to the electrospray Ion-Max source of the mass spectrometer that was operated in MRM mode. The settings of the ion source were as follows: needle voltage, +4.5 kV; sheath gas pressure, 30 (arbitrary units); auxiliary gas pressure, 3 (arbitrary units), and capillary temperature, 250°C. Additionally, to overcome the possible reduction of the signal intensity due to perfluorinated carboxylic acid use, we added 0.05% (v/v) FA in methanol at 0.2 ml/min as the post-column sheet flow. The LC/MS system, data acquisition and processing were managed by the Xcalibur software (v. 2.1, Thermo Scientific).

### Statistical Analysis

All of the data obtained are presented as mean and standard error of the mean (SEM) or in case of the lack of normal distribution as median and range. Statistical tests were done using STATISTICA 10 (Stat Soft Inc., USA) or GraphPad Prism 5 (GraphPad Software, Inc., CA, USA) software. Non-parametric test (Kruskal–Wallis test) or parametric test (one-way ANOVA followed by Tukey’s *post hoc* test) were performed. Pearson or Spearman correlation coefficient tests were used to assess dependence between two parameters. Statistical significance was defined as *p* < 0.05.

## Results

### Effects of MNA and Perindopril Treatment on Endothelium-dependent Vasomotor Response and Changes in Endothelial Permeability in ApoE/LDLR^-/-^ Mice *In Vivo*

**FIGURE 2:**
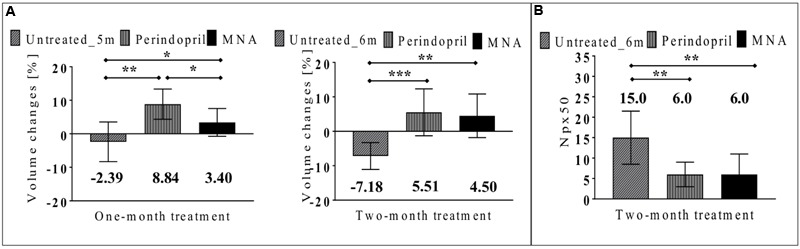
**Effects of MNA and ACE-I (perindopril) treatment on endothelial function and permeability *in vivo* in ApoE/LDLR^-/-^ mice.** Changes in end-diastolic volume of BCA 25 min after Ach administration **(A)** and number of pixels around BCA, for which T_1_ had changed more than 50% after CA administration (Npx50) **(B)** in non-treated ApoE/LDLR^-/-^ mice (columns with diagonal lines) and in ApoE/LDLR^-/-^ mice treated with MNA (black columns) or perindopril (columns with vertical lines) for one month (Untreated_5m *n* = 8, perindopril *n* = 5, MNA *n* = 6) or two months (Untreated_6m *n* = 8, perindopril *n* = 9, MNA *n* = 7). Statistics: Kruskal–Wallis test, ^∗^*p* < 0.05, ^∗∗^*p* < 0.01, ^∗∗∗^*p* < 0.001.

In non-treated ApoE/LDLR^-/-^ mice, injection of Ach (16.6 mg/kg given i.p. in the volume of 50 μl) resulted in vasoconstriction of BCA. Endothelium-dependent response of BCA after Ach administration amounted to –2.39 and –7.18% for 5-month-old and 6-month-old ApoE/LDLR^-/-^ mice, respectively. In mice treated with MNA or perindopril for a 1-month period (**Figure 2A**), endothelium-dependent response improved and an increase in volume of BCA after Ach injection was observed (by about 9 and 3%, respectively). Treatment of ApoE/LDLR^-/-^ mice for 2 months with MNA or perindopril further improved endothelium-dependent vasodilation as well as decreased endothelial permeability (**Figure 2B**). The number of pixels around BCA, for which T_1_ had changed more than 50% after CA administration (Npx50) was smaller in mice treated with MNA and perindopril than in non-treated ApoE/LDLR^-/-^ mice (Npx50 was 6.0, 6.0, and 15.0, respectively, **Figure 2B**). Interestingly, there was a significant correlation between endothelium-dependent response of BCA and Npx50 (**Table 1**).

**Table 1:** Correlation between parameters of functional and biochemical endothelial profiling *in vivo*.

The pair of parameters	R Spearman	*p*
BCA volume changes and Npx50	–0,668739	0,000668
BCA volume changes and L-arginine/ADMA ratio	0,765473	0,000000
BCA volume changes and Ang-(1–7)/Ang II ratio	0,710365	0,000001
Npx50 and L-arginine/ADMA ratio	–0,770589	0,000070
Npx50 and Ang-(1–7)/Ang II ratio	–0,639038	0,002420

### Effects of MNA and Perindopril Treatment on Plasma Ang Profile

**FIGURE 3:**
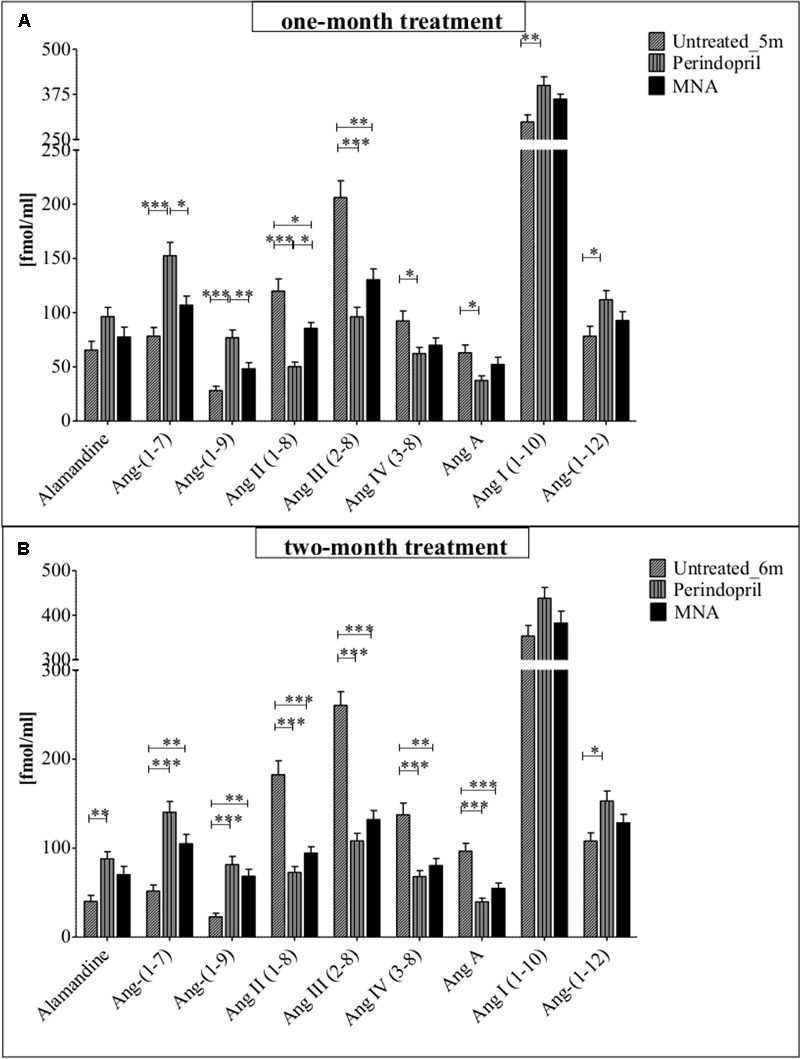
**Effects of MNA and ACE-I (perindopril) treatment on plasma Ang profile in ApoE/LDLR^-/-^ mice.** Plasma Ang profile in non-treated ApoE/LDLR^-/-^ mice (columns with diagonal lines) and in ApoE/LDLR^-/-^ mice treated with MNA (black columns) or perindopril (columns with vertical lines) for one month (**A**: Untreated_5m *n* = 6, perindopril *n* = 5, MNA *n* = 5) or two months (**B**: Untreated_6m *n* = 6, perindopril *n* = 8, MNA *n* = 7). Statistics: one-way ANOVA followed by Tukey’s *post hoc* test (normality was assessed using the Kolmogorov–Smirnov test); ^∗^*p* < 0.05, ^∗∗^*p* < 0.01, ^∗∗∗^*p* < 0.001.

The pattern of changes in plasma Ang profile of ApoE/LDLR^-/-^ mice treated for one month with MNA was largely convergent with those observed in plasma of mice treated with perindopril (as compared to non-treated animals), and involved an increase in concentration of Ang-(1–7) [by 36% (MNA), 95% (perindopril)], alamandine [by 18% (MNA), 47% (perindopril)], and Ang-(1–9) [by 72% (MNA), 173% (perindopril)] in comparison to untreated ApoE/LDLR^-/-^ mice (**Figure 3A**). At the same time, the level of Ang II and other pathological effectors of the RAS (Ang III, IV, A) significantly decreased. The most pronounced differences were observed in the content of Ang II and its N-terminally truncated metabolite – Ang III, the level of which nearly halved as compared to the control, untreated animals [from 119.8 ± 11.3 to 85.5 ± 5.4 (MNA) and to 50.2 ± 4.1 (perindopril) fmol/ml for Ang II, from 206.2 ± 15.5 to 130.4 ± 10.0 (MNA), and to 96.2 ± 8.7 (perindopril) fmol/ml for Ang III, respectively]. These changes were accompanied by a slight increase in the content of precursor peptides: Ang I [by 21% (MNA) and 34% (perindopril) versus non-treated mice] and Ang-(1–12) [by 18% (MNA) and 43% (perindopril) as compared to control animals]. Activation of counter-regulatory ACE2/Ang-(1–7)/Ang-(1–9) axis with concomitant inhibition of ACE/Ang II pathway was further highlighted after two months of treatment with MNA or perindopril whereby the beneficial effect of MNA treatment was even more similar to that exerted by perindopril (**Figure 3B**). Interestingly, there was a significant correlation between Ang-(1–7)/Ang II ratio and endothelium-dependent response of BCA or Npx50 (**Table 1**).

### Effects of MNA and Perindopril Treatment on L-Arg/ADMA Ratio in Plasma

**FIGURE 4:**
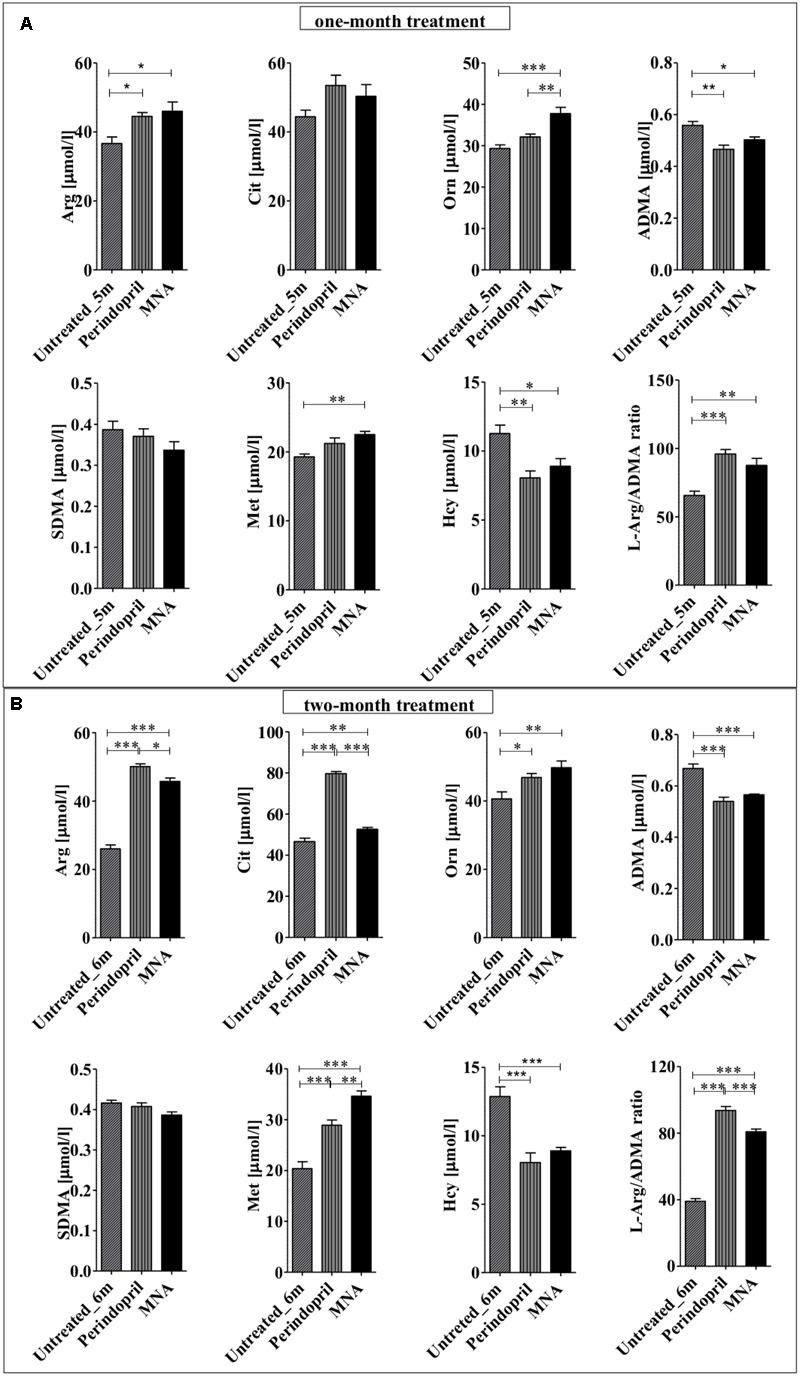
**Effects of MNA and ACE-I (perindopril) treatment on L-arginine/ADMA ratio in plasma in ApoE/LDLR^-/-^ mice.** Plasma concentration of L-Arg, its precursors (L-Cit, L-Orn), ADMA, SDMA, Met, Hcy as well as L-Arg/ADMA ratio in non-treated ApoE/LDLR^-/-^ mice (columns with diagonal lines) and in ApoE/LDLR^-/-^ mice treated with MNA (black columns) or perindopril (columns with vertical lines) for one month (**A**: Untreated_5m *n* = 6, perindopril *n* = 5, MNA *n* = 5) or two months (**B**: Untreated_6m *n* = 6, perindopril *n* = 8, MNA *n* = 7). Statistics: one-way ANOVA followed by Tukey’s *post hoc* test (normality was assessed using the Kolmogorov–Smirnov test); ^∗^*p* < 0.05, ^∗∗^*p* < 0.01, ^∗∗∗^*p* < 0.001.

Endothelial function is critically dependent on eNOS activity and consequently adequate NO bioavailability. Treatment with both MNA and perindopril was associated with alterations in amino acid metabolism engaged in NO generation. Plasma concentration of L-Arg that serves as a substrate for the formation of NO by the NOS enzymes was elevated after just one month of treatment with both MNA (by 25%) and perindopril (21%) as compared to untreated ApoE/LDLR^-/-^ mice (**Figure 4A**). Furthermore, the plasma concentration of precursors of L-Arg (L-citrulline and L-ornithine) was increased in parallel. These changes were accompanied by a decrease in methylated Arg concentration. After 1 month of treatment, ADMA that is perceived as an endogenous inhibitor of all NOS enzymes (including eNOS) was diminished by 10% (MNA) and 17% (perindopril), respectively (in comparison to non-treated animals). Two months of pharmacotherapy led to a further decline in the plasma concentration of ADMA [by 15% (MNA) and by 20% (perindopril), respectively] (**Figure 4B**). Plasma concentration of SDMA, recognized as a competitive inhibitor of cellular L-Arg transport was also decreased after 1 and 2 months of treatment with MNA or perindopril, but these changes did not reach statistical significance. Moreover, reduced concentration of methylated Arg was associated with an increase in the content of Met and concomitant decrease in the level of Hcy. Consequently, plasma L-Arg/ADMA ratio reflecting NO bioavailability was clearly elevated after 1 month of treatment with both MNA (by 34%) and perindopril (by 46%), and this effect was further maintained after 2 months of treatment. Interestingly, there was a significant correlation between L-arginine/ADMA ratio and endothelium-dependent response of BCA or Npx50 (**Table 1**).

## Discussion

In the present work, we profiled endothelial activity of MNA in comparison with perindopril (ACE-I) using functional 3D MRI–based *in vivo* assays of endothelial function in parallel to biochemical measurements of ACE/Ang II-ACE2/Ang-(1–7) balance, and L-Arg/ADMA ratio in plasma. We demonstrated *in vivo* in ApoE/LDLR^-/-^ mice, that treatment with MNA or perindopril resulted in the reversal of Ach-induced vasoconstriction response to vasodilatation and a concomitant decrease in endothelial permeability. Functional improvement of endothelial function by MNA or perindopril treatment was in both cases associated with the inhibition of ACE/Ang II axis and parallel activation of ACE2/Ang-(1–7) axis as evidenced by a fall in plasma concentration of Ang II, and its N-terminally truncated metabolites (Ang III and IV) and an increase in Ang-(1–9) and Ang-(1–7) plasma concentration. Furthermore, MNA and perindopril treatment resulted in an increase in L-arginine/ADMA ratio compatible with improvement of NO-dependent endothelial function. Altogether, our results suggest that MNA displayed a similar pattern of vasoprotective activity as perindopril, a representative of ACE-Is, and both compounds affected ACE/Ang II-ACE2/Ang-(1–7) balance and L-Arg/ADMA ratio that could contribute to their vasoprotective effects. Given that correction of endothelial function by ACE-Is contributes significantly to their clinical benefits and their anti-inflammatory, anti-thrombotic, anti-diabetic, and vasoprotective actions reported in numerous experimental and clinical papers (Chłopicki and Gryglewski, 2005), the findings of endothelial profile of MNA being similar to perindopril in *in vivo* settings bring important novel perspectives to understanding the therapeutic efficacy of MNA reported previously (Gebicki et al., 2003; Wozniacka et al., 2005; Chlopicki et al., 2007; Bartuś et al., 2008; Bryniarski et al., 2008; Brzozowski et al., 2008; Watała et al., 2009; Sternak et al., 2010; Przyborowski et al., 2015; Blazejczyk et al., 2016; Jakubowski et al., 2016; Mateuszuk et al., 2016).

In this study, endothelial function *in vivo* assessment was performed in BCA, in response to intraperitoneal administration of Ach. Importantly, endothelium-dependent response was independent of the effect of Ach on the heart and respiration (Bar et al., 2016). Undoubtedly, the important advantage of the use of MRI-based method is a retrospective reconstruction of images from 3D data sets, which allows for assessment of response of the entire vessel instead of only one fragment of the vessel. Moreover, the method provides the possibility of accurate positioning after experiment, whereby the effects of endothelial pharmacotherapy can be assessed, even in small vessels such as BCA, across various cross-sections to be located in planes perpendicular to the vessel. The 3D gradient echo sequence allows the avoidance of precise slice positioning during measurements, as arbitrary re-slicing during post-processing is possible with shorter time of the measurements. Importantly, in the present work an MRI-based method to assess endothelial permeability changes was also employed. We assessed the number of pixels (around the BCA) for which T_1_ had changed more than 50% (Npx50), as described previously (Bar et al., 2016). Npx50 parameter allows avoiding precise determination of ROI, what is difficult to establish objectively. Finding the pixels for which T_1_ had changed more than 50%, allows for operator-independent assessment of the ROI around the vessel. Analysis of changes in endothelial permeability represents an important feature of endothelial dysfunction, and its measurements seem a valuable test to analyze endothelial state *in vivo*. When endothelial permeability increases, leaky intercellular junctions of endothelium allow access to low-density lipoprotein particles and transmigration of leukocytes that are critical in atherosclerotic plaque formation (Kao et al., 1995). Moreover, albumin-binding CA can also accumulate in vessel walls (Lobbes et al., 2009; Pedersen et al., 2011), and this results in shortening of the T_1_ in the vessel wall enabling MRI-based detection, and Npx50-based operator-independent assessment of endothelial permeability (Bar et al., 2016). Importantly, in the present work, analysis of endothelial-dependent response by Ach as well as endothelial permeability based on Npx50 displayed significant negative correlation and gave concordant results showing improvement after MNA and perindopril treatment. Experiments carried out in this paper support the notion that MRI-based technique based on the retrospectively self-gated 3D IntraGate^®^ FLASH sequence (Bar et al., 2016) is useful for monitoring the efficacy of endothelium-targeted therapy in murine models of diseases *in vivo* and seems far superior to ultrasound (Cavalcante et al., 2011).

In non-treated ApoE/LDLR^-/-^ mice, injection of Ach resulted in paradoxical vasoconstriction, what was most likely due to a smooth muscle muscarinic receptor-dependent response, as also shown for human arteries with endothelial dysfunction (Ludmer et al., 1986). Treatment with MNA or perindopril resulted in shifting the Ach response to a vasodilatation, which indicates improvement in endothelial function. Comparing results of changes in BCA volume after acetylcholine administration in 5-month-old C57BL/6J mice (9.3%) published in our previous paper (Bar et al., 2016), to ApoE/LDLR-/- mice after two-month treatment with MNA (4.5%) or perindopril (5.5%) performed in present study, it seems that either treatment considerably improved the impaired endothelium-dependent response. It is worth noting that, after one-month treatment effects of perindopril seemed to be more pronounced as compared with the effect of MNA, but this difference disappeared after two months of treatment. Improvement in endothelial permeability was also similar in magnitude for MNA and perindopril after two months of treatment, supporting the similar pattern of endothelial response to MNA given in a dose of 100 mg/kg b.w./day in comparison to perindopril given in a dose of 10 mg/kg b.w./day.

An important finding of the present study was the demonstration that improvement of endothelial function by MNA and perindopril treatment, evidenced by MRI-based assays, was in both cases associated with the inhibition of the classical RAS pathway – ACE/Ang II/AT_1_R and concomitant activation of counter-regulatory RAS pathway – ACE2/Ang-(1–7)/Mas. Numerous previous studies clearly indicate that the RAS plays a critical role in the initiation and progression of atherosclerosis with endothelial dysfunction as its early event, and that the balance between two major axes of RAS, i.e., ACE/Ang II and ACE2/Ang-(1–7), is a key determinant of RAS beneficial vs. detrimental effects (Wang et al., 2013; Olkowicz et al., 2015; Cahill and Redmond, 2016). Ang II is considered as a pro-atherosclerotic mediator which causes vasoconstriction, regulates adhesion molecule (ICAM-1, VCAM-1, P-selectin) expression as well as chemokine, cytokine, and growth factor secretion within the arterial wall (Jiang et al., 2014). On the other hand, ACE2, through its product Ang-(1–7) displays anti-atherosclerotic properties *in vivo*, including decreasing inflammation and oxidative stress, and inhibiting inflammatory cell infiltration. The protective effect of ACE2, mainly distributed in ECs, VSMCs, and macrophages, was repeatedly demonstrated. Overexpression of ACE2 by gene transfer attenuated the progression of atherosclerotic lesions when the ACE2 gene was transferred 4 weeks after injury in a rabbit model of atherosclerosis (Dong et al., 2008). Similarly, overexpression of ACE2 in ApoE-deficient mice decreased atherosclerotic lesion size within the aortic sinus as compared to control mice (Lovren et al., 2008). In contrast, ACE2-deficiency in LDLR^-/-^ as well as ApoE^-/-^ genetic background mice increased the development of atherosclerosis (Thomas et al., 2010; Thatcher et al., 2011).

Our results not only confirmed that perindopril-induced a vasoprotective shift in the balance of ACE/Ang II and ACE2/Ang-(1–7), but also underscored that the profile of MNA is similar to that exerted by perindopril. Zhuo et al. (2002) have recently demonstrated that the clinical benefits of chronic ACE inhibition with perindopril, in addition to the blockade of systemic and tissue Ang II formation, may be in part due to elevated bradykinin levels, which in turn lead to increased NO bioavailability. Bradykinin was shown to be the major mediator of ACE-I-induced anti-thrombotic effects (Gryglewski et al., 2003, 2005; Chłopicki and Gryglewski, 2005). We did not study here the relative contribution of Ang II–dependent and bradykinin–dependent pathway to perindopril-induced improvement in NO-dependent function, but obviously it cannot be excluded that both pathways may be involved in perindopril-induced improvement in endothelial function. Whether the same mechanisms contribute to MNA- induced effects remains to be established. Interestingly, in our previous studies describing anti-atherosclerotic effects of MNA, an improvement in NO–dependent endothelial function, observed in *ex vivo* vascular preparations was also associated with activation of PGI_2_–dependent function (Mateuszuk et al., 2016). Altogether, the results presented here suggest that MNA- induced shift in the balance of ACE/Ang II and ACE2/Ang-(1–7) toward the vasoprotective axis may contribute to the improvement of endothelial function by MNA or alternatively may constitute an important pathophysiological sign of improved endothelial function.

We also demonstrated that MNA and perindopril treatment resulted in an increase in L-arginine/ADMA ratio that was compatible with improvement of NO-dependent endothelial function. Given the fact, that circulating level of ADMA adds independent prognostic information with regard to cardiovascular risk beyond that obtained from classical risk factors and novel biomarkers (Schnabel et al., 2005) this finding is of importance. In fact, plasma concentrations of ADMA have been found to be significantly elevated in patients with arteriosclerosis, as well as in those with coronary risk factors, such as hypertension or hypercholesterolemia, and marked increase in ADMA level has been associated with impaired endothelium-dependent NO-mediated vasodilatation (Böger et al., 1997, 1998; Surdacki et al., 1999; Böger, 2003) that may be linked to decreased activity of y+LAT as the major export pathway for the ADMA (Closs et al., 2012). Interestingly, Suda et al. (2004) have recently reported that the long-term vascular effects of ADMA are not solely mediated by inhibition of endothelial NO synthesis but also can lead to direct upregulation of ACE expression and increased oxidative stress through AT_1_ receptor activation. Moreover, they have proposed that inhibition of NO synthesis appears primarily as a result of short-term action of ADMA; as long as metabolites of ADMA accumulate at higher concentrations in blood vessels, eNOS-independent mechanisms predominate. Involvement of multiple mechanisms in vascular effects of ADMA, other than simple inhibition of endothelial NO synthesis, including also upregulation of ACE, remains to be clarified. In a recent work of Jiang et al. (2016) it was reported that MNA attenuated atherogenesis *via* modulation of the ADMA-DDAH pathway in ApoE-deficient mice. The beneficial effect of MNA treatment was associated with the upregulation of the DDAH2 enzyme activity in endothelium *via* the mitigation of hypermethylation in the promoter region of the enzyme. These results seem consistent with our observations showing the reduction in plasma ADMA concentration as well as Hcy concentration that was linked to an improved endothelium-dependent vascular relaxation after MNA. Interestingly, Huang et al. (2015) showed that Hcy synthesized in endothelium (after Met administration) affected activity of ACE by direct homocysteinylation of its amino- and/or sulfhydryl- moieties. This modification enhanced ACE reactivity toward Ang II and consequently led to NADPH oxidase-superoxide-dependent endothelial dysfunction. In that context, treatment with MNA and perindopril attenuates various apparently interlinked biochemical changes closely associated with functional phenotype of endothelial dysfunction such as excessive activation of ACE, elevation of ADMA and Hcy that can all contribute to vasoprotective action of these compounds resulting in the improvement of endothelial function reported here *in vivo* using functional and biochemical measurements.

Indeed, there was a significant positive correlation between endothelium-dependent response of BCA after acetylcholine administration and the two major biochemical parameters measured here [L-arginine/ADMA ratio and Ang-(1–7)/Ang II]. In turn, since the improvement of endothelial dysfunction was associated with a decrease in endothelial permeability, correlation between Npx50 and biochemical parameters [L-arginine/ADMA ratio and Ang-(1–7)/Ang II] had negative character. Similar correlations between ADMA concentration and Ach- or flow-induced vasodilatation have also been demonstrated previously in monkeys with hypercholesterolemia, using endothelium-dependent vascular function *ex vivo* assessment (Böger et al., 2000) as well as in humans with hypercholesterolemia using ultrasonography (Böger et al., 1998).

Effects of treatment with MNA and perindopril on atherosclerotic plaque burden were not assessed in this study, but it was reported previously that perindopril treatment inhibited the development of atherosclerotic lesions in diabetic ApoE-deficient mice (Candido et al., 2002), while anti-atherosclerotic effect of MNA was described in apolipoprotein E (ApoE)/low-density lipoprotein receptor (LDLR)-deficient mice (Mateuszuk et al., 2016). Given the pathophysiological role of endothelial dysfunction in atherogenesis, effects of MNA or perindopril on endothelial function could contribute to the anti-atherosclerotic action of these compounds.

## Conclusion

Using functional 3D MRI-based assays of endothelial function and biochemical measurements of ACE/Ang II, ACE2/Ang-(1–7) balance and L-Arg/ADMA ratio in plasma, we demonstrated significant vasoprotective effects of MNA (100 mg/kg b.w./day), which were comparable with that afforded by perindopril (10 mg/kg b.w./day), a well-known ACE inhibitor. Although effects of MNA were less pronounced as compared with perindopril after 1-month treatment, after 2-month treatment, either treatment modality resulted in a comparable effect including reversal of Ach-induced vasoconstriction response to vasodilatation and a concomitant decrease in endothelial permeability. Furthermore, functional improvement of endothelial function by MNA or perindopril treatment was in both cases associated with the inhibition of ACE/Ang II axis, the parallel activation of ACE2/Ang-(1–7) axis, and an increase in L-Arg/ADMA ratio in plasma. Given that improvement in endothelial function by ACE-Is contributes to their therapeutic efficacy (Chłopicki and Gryglewski, 2005), presented in the current study findings of endothelial profile of MNA being similar to ACE-I (perindopril) in *in vivo* settings bring important novel perspectives to understand therapeutic efficacy of MNA (Gebicki et al., 2003; Wozniacka et al., 2005; Chlopicki et al., 2007; Bartuś et al., 2008; Bryniarski et al., 2008; Brzozowski et al., 2008; Watała et al., 2009; Sternak et al., 2010; Przyborowski et al., 2015; Blazejczyk et al., 2016; Jakubowski et al., 2016; Mateuszuk et al., 2016). Moreover, our approach for the *in vivo* assessment of endothelial phenotype based on a retrospectively self-gated 3D gradient-echo sequence, MRI-based technique, concomitant with the biochemical assays of two important systems of endothelial regulation, ACE/Ang-II-ACE2/Ang-(1–7) and L-Arg/ADMA, proves to be a useful tool for the *in vivo* endothelial profiling of compounds to demonstrate convincingly their beneficial effects on endothelial function.

## Author Contributions

Conceived and designed the study: AB and SC. Performed the study: AB, MO, UT, EK, and KJ. Analyzed the data: AB and MO. Provided the analytical tools: RS, TS. Drafted the manuscript: AB, MO, and SC. All authors have corrected or have approved the final version of the manuscript.

## Conflict of Interest Statement

SC is a coinventor of the patent on the use of quaternary pyridinium salts as vasoprotective agents. The other authors declare that the research was conducted in the absence of any commercial or financial relationships that could be construed as a potential conflict of interest.

## Abbreviations

ACE: angiotensin-converting enzyme
ACE-I: angiotensin-converting enzyme inhibitor
Ach: acetylcholine
ACN: acetonitrile
ADMA: asymmetric dimethylarginine
Ang: angiotensin
ApoE/LDLR^-/-^: atherosclerotic mice
Arg: arginine
BCA: brachiocephalic artery
CA: contrast agent
COX-2: cyclooxygenase 2
CS: calibration standards
DDAH: dimethylarginine dimethylaminohydrolase
ECs: endothelial cells
eNOS: endothelial nitric oxide synthase
FA: formic acid
Hcy: homocysteine
HFD: high-fat diet
IL-4: interleukin-4
IS: internal standard
LC-MS/MS: liquid chromatography-tandem mass spectrometry
Met: L-methionine
MNA: 1-methylnicotinamide
MRI: magnetic resonance imaging
MRM: multiple reaction monitoring mode
NA: nicotinamide
NFPA: nonafluoropentanoic acid
NNMT: nicotinamide N-methyltransferase
NO: nitric oxide
Npx50: number of pixels for which T_1_ had changed more than 50% after contrast agent administration
PGI_2_: prostacyclin
QC: quality control samples
RAS: renin-angiotensin system
ROI: region of interest
ROS: reactive oxygen species
RS: real samples
SDMA: symmetric dimethylarginine
VFA: Variable Flip Angle technique
VSMCs: vascular smooth muscle cells
T_1_: relaxation time
TNF-α: tumor necrosis factor-α
