# Supplementary figures and images for: Functional and Biochemical Endothelial Profiling In Vivo in a Murine Model of Endothelial Dysfunction; Comparison of Effects of 1-Methylnicotinamide and Angiotensin-converting Enzyme Inhibitor
Source: Front Pharmacol. 2017 Apr 10;8:183. doi: 10.3389/fphar.2017.00183 (PMC5385379; doi:10.3389/fphar.2017.00183)

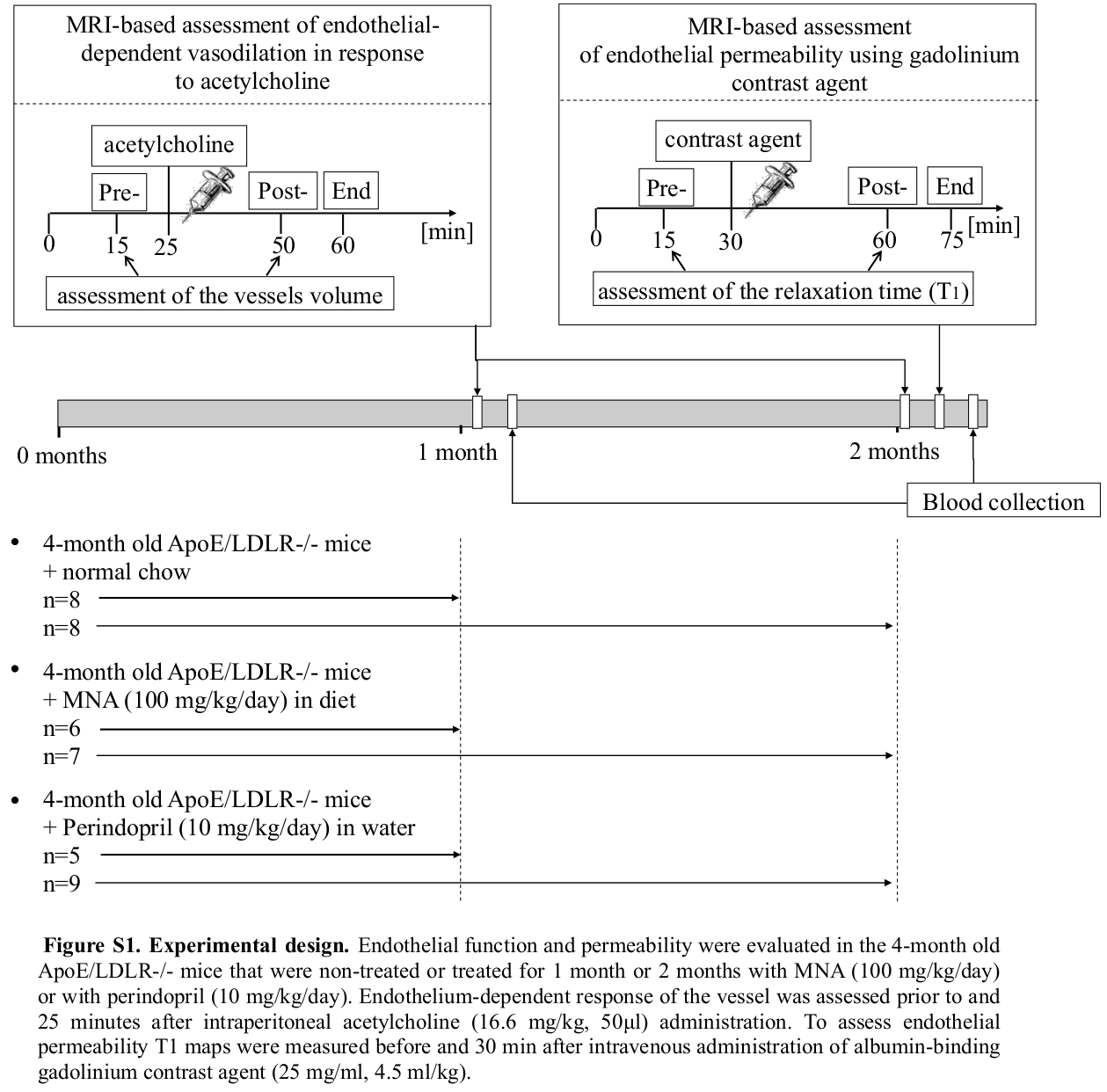

Supplement: Supplementary file 1 [file Image_1.tif]
